# Consumer Views on Privacy Protections and Sharing of Personal Digital Health Information

**DOI:** 10.1001/jamanetworkopen.2023.1305

**Published:** 2023-03-02

**Authors:** Ravi Gupta, Raghuram Iyengar, Meghana Sharma, Carolyn C. Cannuscio, Raina M. Merchant, David A. Asch, Nandita Mitra, David Grande

**Affiliations:** 1 Johns Hopkins University School of Medicine, Baltimore, Maryland; 2 Hopkins Business of Health Initiative, Johns Hopkins University, Baltimore, Maryland; 3 Center for Health Services and Outcomes Research, Johns Hopkins Bloomberg School of Public Health, Baltimore, Maryland; 4 Wharton School, University of Pennsylvania, Philadelphia; 5 Perelman School of Medicine, Division of General Internal Medicine, Department of Medicine, University of Pennsylvania, Philadelphia; 6 Perelman School of Medicine, Department of Family Medicine and Community Health, University of Pennsylvania, Philadelphia; 7 Leonard Davis Institute of Health Economics, University of Pennsylvania, Philadelphia; 8 Perelman School of Medicine, Department of Emergency Medicine, University of Pennsylvania, Philadelphia; 9 Perelman School of Medicine, Department of Biostatistics, Epidemiology and Informatics, University of Pennsylvania, Philadelphia

## Abstract

**Importance:**

Digital health information has many potential health applications, but privacy is a growing concern among consumers and policy makers. Consent alone is increasingly seen as inadequate to safeguard privacy.

**Objective:**

To determine whether different privacy protections are associated with consumers’ willingness to share their digital health information for research, marketing, or clinical uses.

**Design, Setting, and Participants:**

This 2020 national survey with an embedded conjoint experiment recruited US adults from a nationally representative sample with oversampling of Black and Hispanic individuals. Willingness to share digital information across 192 different scenarios reflecting the product of 4 possible privacy protections, 3 uses of information, 2 users of information, and 2 sources of digital information was evaluated. Each participant was randomly assigned 9 scenarios. The survey was administrated between July 10 and July 31, 2020, in Spanish and English. Analysis for this study was conducted between May 2021 and July 2022.

**Main Outcomes and Measures:**

Participants rated each conjoint profile on a 5-point Likert scale measuring their willingness to share their personal digital information (with 5 indicating the most willingness to share). Results are reported as adjusted mean differences.

**Results:**

Of the 6284 potential participants, 3539 (56%) responded to the conjoint scenarios. A total of 1858 participants (53%) were female, 758 (21%) identified as Black, 833 (24%) identified as Hispanic, 1149 (33%) had an annual income less than $50 000, and 1274 (36%) were 60 years or older. Participants were more willing to share health information with the presence of each individual privacy protection, including consent (difference, 0.32; 95% CI, 0.29-0.35; *P* < .001), followed by data deletion (difference, 0.16; 95% CI, 0.13-0.18; *P* < .001), oversight (difference, 0.13; 95% CI, 0.10-0.15; *P* < .001), and transparency of data collected (difference, 0.08; 95% CI, 0.05-0.10; *P* < .001). The relative importance (importance weight on a 0%-100% scale) was greatest for the purpose of use (29.9%) but when considered collectively, the 4 privacy protections together were the most important (51.5%) factor in the conjoint experiment. When the 4 privacy protections were considered separately, consent was the most important (23.9%).

**Conclusions and Relevance:**

In this survey study of a nationally representative sample of US adults, consumers’ willingness to share personal digital health information for health purposes was associated with the presence of specific privacy protections beyond consent alone. Additional protections, including data transparency, oversight, and data deletion may strengthen consumer confidence in sharing their personal digital health information.

## Key Points

**Question:**

Are privacy protections, including consent, transparency of collected data with the consumer, regulatory oversight over data use, and ability to delete data, associated with consumers’ willingness to share their digital health information?

**Findings:**

In this survey study of 3539 US adults, conjoint analyses revealed that a combination of privacy protections including consent, consumer access to data collected from them, ethical and regulatory oversight, and ability to delete data together were associated with higher consumer willingness to share their digital health data.

**Meaning:**

Results of this study suggest that strengthening consent as a primary privacy protection and adding protections including data transparency, regulatory oversight, and ability to delete data may increase consumer trust and thereby support socially beneficial uses of digital health data.

## Teaser

This survey study uses a nationally representative population to determine consumers’ perception of the relative importance of specific privacy protections, including consent, data transparency, regulatory oversight, and ability to delete previously collected personal data in various uses of digital health data.

## Introduction

Interactions with the health care system and use of wearable devices, social media, telephone apps, and retail generate vast amounts of digital data reflecting personal health. These data can lead to meaningful social benefits, such as identifying individuals’ mental health concerns through social media,^1,2,3^ building algorithms to estimate risk of developing conditions such as dementia^4^ and cardiovascular disease,^5^ and tracking COVID-19 infections.^6^ The growing collection of digital health information and blurred lines between health and nonhealth data also raise privacy and security concerns that are in tension with benefits. The Supreme Court’s decision in *Dobbs v Jackson Women’s Health*,^7^ for example, has elevated concerns that digital health information from menstrual period tracker apps and website purchases may reveal sensitive reproductive health data.^8^ The 1996 Health Insurance Portability and Accountability Act (HIPAA) offers privacy protections only for health data and for certain health entities, which excludes most internet data and large technology firms.^9,10^

Protection of consumer privacy has relied heavily on a model of consent. Prior literature^11,12^ has demonstrated the shortcomings of consent in research protocols and clinical care given the complexity of understanding required from patients to make informed decisions, particularly with the growing involvement of large technology companies.^13^ Several reasons explain this inadequacy, including the inability to estimate future uses of data at the time of collection; dense and convoluted company privacy policies that can change without consumer notice; abrogation of companies’ responsibility after attaining consent; and shifting of an impossible burden onto individuals to understand the policies, make choices, and oversee the continued use of their personal data.^14^ In other cases, such as health data sharing with third parties, consent may be absent altogether.^15^

The proliferation of health data from consumer digital interactions and sophisticated data science methods thus requires new approaches to health information privacy beyond consent.^13,16,17^ To our knowledge, no studies have systematically examined how privacy protections increase consumer willingness to share their digital health information. We studied a nationally representative population to determine consumer perceptions of the relative importance of specific privacy protections derived from the fair information practice principles and approaches in other nations,^18,19,20^ including consent, data transparency, regulatory oversight, and ability to delete previously collected personal data in various uses of digital health data.

## Methods

We used the web-enabled Ipsos KnowledgePanel to recruit participants for this cross-sectional survey study, as previously described.^21^ Ipsos is a probability-based panel designed to be representative of the US population, with participants recruited using address-based sampling methods.^22^ At the time of joining the panel, participants were asked to complete a general informed consent process followed by participants self-reporting key demographic characteristics including race and ethnicity using the US Census Bureau categories. We assessed race and ethnicity in this study given known racial and ethnic differences in concerns about privacy and historical distrust in biomedical research,^23,24,25^ with oversampling of Black and Hispanic individuals. We also ascertained participants’ political ideology, given that political views have been associated with trust in various uses of consumer digital data.^26^

The survey was administrated between July 10 and July 31, 2020, in Spanish and English. Analysis for this study was conducted between May 2021 and July 2022. All data received by the study team were deidentified. This study was reviewed and deemed exempt from the need for informed consent by the University of Pennsylvania Institutional Review Board based on the minimal risk of the research and use of deidentified data. This study followed reporting guidelines and ethical conducts of public opinion as well as survey research as defined by American Association for Public Opinion Research (AAPOR) reporting guideline.

### Survey Administration and Conjoint Scenarios

We used conjoint analysis to measure consumer willingness to share their digital health information. Conjoint analysis is widely used in marketing to assess consumer preferences.^27^ Participants rate descriptions of items or circumstances that vary along established dimensions felt to be important (eg, color, price, quality) and statistical models are used to identify the relative contributions of each dimension to the overall rating. In our context, we evaluated 4 digital information use attributes in the scenarios: the information being used (information type), who is using it (user), the purpose of use (use), and the privacy protections present (privacy protection). The experimental design included 192 possible scenarios reflecting a full factorial design of 2 users, 2 information types, 3 uses, and the absence or presence of 4 different privacy protections (Table 1). The survey instrument was adapted from a prior instrument using conjoint analysis to assess consumer privacy preferences (eMethods in Supplement 1).^28^

**Table 1.** Conjoint Design Attributes

Design element	Text presented to respondents
**User**	
University hospital	Information used by a university hospital.
Digital technology company	Information used by a digital technology company.
**Source**	
Mobile telephone	Information about places people visit from apps or software on their telephone.
Electronic health records	Information about people’s health from their electronic medical records.
**Use**	
Research	Published their results in a medical journal so that doctors could learn how to improve diabetes care.
Marketing	Used this information to develop a marketing campaign to double the number of people taking a diabetes medication.
Clinical	Used this information to recommend changes to patients to improve their diabetes care.
**Privacy protections**	
Consent	People were asked permission for their information to be used.
Data transparency	People were able to view the data that were collected from them.
Oversight	A group of experts determined that personal privacy would be well-protected.
Data deletion	People could request that their data be erased at any time.

The conjoint attributes and levels were selected based on qualitative interviews with consumers and subject matter experts.^29,30^ We conducted cognitive interviews^31^ to evaluate the survey instrument for clarity and participant comprehension prior to administration. Participants were asked to evaluate 9 scenarios (ie, profiles) randomly selected from the 192 total. The scenarios were presented in the context of diabetes care, meaning that all scenarios reflected reusing data for the purposes of reducing the risk of diabetes. Participants rated each scenario on a 5-point Likert scale assessing their willingness to share their information of 1 (definitely would share) to 5 (definitely would not share). We reversed the scale in analyses, with 1 indicating least willingness to share and 5 most willingness to ease interpretation.

Scenarios were constructed using 2 different information types chosen to reflect those relevant for consumers’ health: information about places people visit from apps or software on their telephone and health information from electronic health records. There were 2 different users of the participant’s data: a university hospital and a digital technology company. The 3 possible uses of data included research (published results in a medical journal to help doctors improve diabetes care), clinical (help patients improve their diabetes care), and marketing (develop a marketing campaign to double the number of people taking a diabetes medication).

The scenarios included 4 different privacy protections based on the fair information practice principles originating from 1967 work by Westin^18^ and refined by the Federal Trade Commission in 2000 reflecting consumers’ preferences on the most important protections.^19^ The first was whether the person was asked permission for their data to be used (consent). The other nonconsent privacy protections included whether people could view the data collected from them (transparency), a group of experts determined that personal privacy would be well-protected (oversight), and people could request that their data be erased at any time (deletion).

### Statistical Analysis

Conjoint analysis uses information on how consumers assess trade-offs across attributes to determine the attributes’ relative importance, termed part-worths. In this study, the part-worth utilities for each level of each conjoint attribute were computed using a generalized estimating equation model to account for correlation of responses within participants, under a gaussian distribution and identity link and assuming an independent working correlation structure with robust, empirical standard errors. In these models, positive differences represent more favored levels and negative differences represent less favored levels relative to a baseline level for each attribute. For each attribute, the difference between the maximum and minimum part-worth utilities reflects how important that attribute is in determining the profiles’ attractiveness. Each attribute’s range is normalized by the sum of the ranges across attributes to allow a comparison of the importance across attributes.

Poststratification weights provided by Ipsos were used across the participant sample to account for differential rates of nonresponse and oversampling to reflect the US population. All statistical tests were 2-tailed and with a significance level of .05.

The main attribute of interest was the relative importance of each privacy protection in consumers’ willingness to share their digital health information for additional uses. We conducted additional analyses for second order interactions between combinations of each privacy protection. For 16 representative conjoint scenarios, we determined part-worth utilities. We also assessed interaction effect sizes between each privacy protection and consumer sociodemographic characteristics including race and ethnicity, political ideology, household income, and age. We used Stata version 16 (StataCorp LP) to conduct all analyses.

## Results

Of the 6284 potential participants, 3539 (56%) responded; a total of 1858 participants (53%) were female, 758 (21%) identified as Black, 833 (24%) identified as Hispanic, 1149 (33%) had an annual income less than $50 000, and 1274 (36%) were 60 years or older (Table 2). The participant political ideologies were nearly evenly split among liberal, moderate, and conservative points of view.

**Table 2.** Characteristics of 3539 Participants in the Survey Study

Characteristic	No. (%)
Gender	
Male	1681 (47.5)
Female	1858 (52.5)
Race^a^	
Black	758 (21.4)
White	2524 (71.3)
Other^b^	148 (4.2)
>2 Races	109 (3.1)
Ethnicity^a^	
Hispanic	833 (23.5)
Non-Hispanic	2706 (76.5)
Age group, y	
18-29	427 (12.1)
30-44	837 (23.7)
45-59	1001 (28.3)
≥60	1274 (36.0)
Political ideology	
Liberal	1046 (30.1)
Moderate	1298 (37.3)
Conservative	1136 (32.6)
Annual household income, $	
≤24 999	476 (13.5)
25 000-49 999	673 (19.0)
50 000-99 999	1164 (32.9)
≥100 000	1226 (34.6)

^a^ Participants completed a general informed consent process followed by participant self-reporting race and ethnicity using the US Census Bureau categories.

^b^ Other includes Asian, American Indian, and Hawaiian and Pacific Islander.

Table 3 presents main interaction effect sizes results from the conjoint experiment. Model coefficients represent differences in consumers’ willingness to share their digital health information. The relative importance (importance weight on a 0%-100% scale) was greatest for the purpose of use (29.9%) but when considered collectively, the 4 privacy protections together were the most important (51.5%). When privacy protections were considered separately, consent was the most important among the 4 protections (23.9%).

**Table 3.** Data Attributes and Demographic Characteristics Associated with Willingness to Share Digital Health Information^a^

Data attribute	Difference (95% CI)	*P* value
Intercept	3.13 (3.00 to 3.26)	<.001
Privacy protection^b^		
Consent	0.32 (0.29 to 0.35)	<.001
Transparency	0.08 (0.05 to 0.10)	<.001
Oversight	0.13 (0.10 to 0.15)	<.001
Data deletion	0.16 (0.13 to 0.18)	<.001
User		
University hospital	1 [Reference]	NA
Digital technology company	−0.17 (−0.20 to −0.14)	<.001
Source		
Places you visit via apps on your telephone	1 [Reference]	NA
Health via EHR	0.08 (0.05 to 0.10)	<.001
Use		
Research	1 [Reference]	NA
Clinical care	−0.09 (−0.12 to −0.06)	<.001
Marketing	−0.40 (−0.44 to −0.37)	<.001
Race^c^		
Black	0.06 (−0.01 to 0.14)	.10
White	1 [Reference]	NA
Other^d^	0.07 (−0.07 to 0.21)	.35
≥2 Races	0.15 (0.00 to .29)	.05
Ethnicity^c^		
Hispanic	0.12 (0.04 to 0.19)	.001
Non-Hispanic	1 [Reference]	NA
Age group, y		
18-29	1 [Reference]	NA
30-44	−0.01 (−0.11 to 0.09)	.83
45-59	−0.16 (−0.26 to −0.06)	.001
≥60	−0.17 (−0.27 to −0.08)	<.001
Political ideology		
Liberal	1 [Reference]	NA
Moderate	−0.16 (−0.23 to −0.10)	<.001
Conservative	−0.26 (−0.33 to −0.19)	<.001
Household income, $		
<24 999	1 [Reference]	NA
25 000-49 999	0.08 (−0.02 to 0.19)	.11
50 000-99 999	−0.03 (−0.12 to 0.07)	.55
>100 000	0.03 (−0.06 to 0.13)	.48

Abbreviations: EHR, electronic health record; NA, not applicable.

^a^ Part-worth utilities from linear generalized estimating equation model.

^b^ Each privacy protection was compared with the absence of that privacy protection. For example, the presence of consent or no consent.

^c^ Participants completed a general informed consent process followed by participant self-reporting race and ethnicity using the US Census Bureau categories.

^d^ Other includes Asian, American Indian, and Hawaiian and Pacific Islander.

Participants were most willing to share health information with the presence of each individual privacy protection, including consent (difference, 0.32; 95% CI, 0.29-0.35; *P* < .001), followed by data deletion (difference, 0.16; 95% CI, 0.13-0.18; *P* < .001), regulatory oversight (difference, 0.13; 95% CI, 0.10-0.15; *P* < .001), and data transparency (difference, 0.08; 95% CI, 0.05-0.10; *P* < .001). We tested second-order interactions between privacy protections, which were generally not significant or small with negative effect sizes (−0.06 or less) with the exception of consent and data deletion (difference, −0.10; 95% CI, −0.15 to −0.05; *P* < .001) (eTable in Supplement 1). Among 16 representative scenarios, compared with a baseline of data being used by university hospitals for research purposes in the absence of any privacy protections, the greatest willingness to share digital health information was when data were used by university hospitals for research purposes in the presence of all 4 privacy protections (3.81; 95% CI, 3.76-3.87; *P* < .001) (Figure). The lowest willingness to share was when the data were used by digital technology companies for marketing purposes in the absence of any privacy protections (2.56; 95% CI, 2.51-2.60; *P* < .001).

**Figure.**
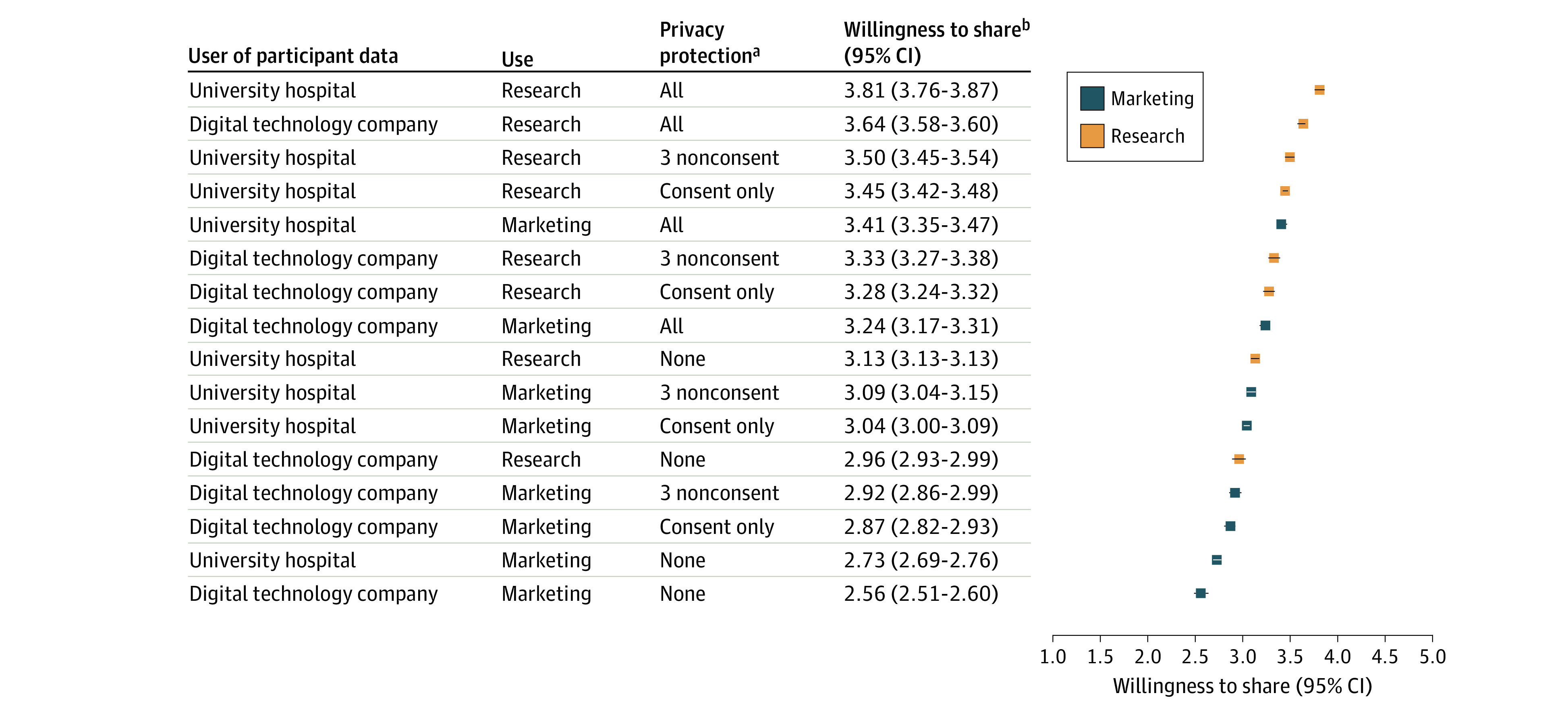
Part-Worth Utilities From Specific Conjoint Scenarios ^a^The 4 privacy protections were consent, data transparency, oversight, and data deletion. ^b^Participants rated each scenario on a 5-point Likert scale assessing their willingness to share their information, with 1 indicating least willingness to share and 5 most willingness.

In comparison with information about places visited from telephone apps, participants were slightly more willing to share health information from personal electronic health records (difference, 0.08; 95% CI, 0.05-0.10; *P* < .001). Compared with a university hospital, participants were less willing to share health information with digital technology companies (difference, −0.17; 95% CI, −0.20 to −0.14; *P* < .001). Relative to digital health information being used for diabetes care research, participants were less willing to share health information for clinical purposes (difference, −0.09; 95% CI, −0.12 to −0.06; *P* < .001) and even less willing for marketing purposes to increase prescriptions of a diabetes medication (difference, −0.40; 95% CI, −0.44 to −0.37; *P* < .001).

In the main interaction effect size model, there were no differences between Black and White respondents in willingness to share health information. Compared with Non-Hispanic respondents, Hispanic respondents were more willing to share health information (difference, 0.12; 95% CI, 0.04-0.19; *P* = .001). Willingness to share health information decreased as age increased. Compared with respondents who self-identified as being liberal, those who self-identified as conservative were less willing to share health information (difference, −0.26; 95% CI, −0.33 to −0.19; *P* < .001). A single model was used to test interactions of each privacy protection and demographic characteristics including race and ethnicity, political ideology, household income, and age (Table 4). Interactions between each privacy protection and demographic characteristics were generally nonsignificant, though requiring consent was a greater factor for non-Hispanic respondents and those earning greater than $100 000 in their willingness to share health information (differences, 0.13 [95% CI, 0.06-0.20] and 0.18 [95% CI, 0.09-0.28], respectively).

**Table 4.** Interaction Effect Size Estimates Between Privacy Protections and Demographic Characteristics^a^

Characteristic	Interaction effect size estimate														
	Main^b^			Consent^c^			Deletion^c^			Transparency^c^			Oversight^c^		
	Difference (95% CI)	*P* value	Difference (95% CI)		*P* value	Difference (95% CI)		*P* value	Difference (95% CI)		*P* value	Difference (95% CI)		*P* value
Protections														
Consent	0.21 (0.09 to 0.34)	.001	NA		NA	NA		NA	NA		NA	NA		NA
Deletion	0.25 (0.13 to 0.36)	<.001	NA		NA	NA		NA	NA		NA	NA		NA
Transparency	0.00 (−0.11 to 0.11)	>.99	NA		NA	NA		NA	NA		NA	NA		NA
Oversight	0.11 (0.00 to 0.22)	.05	NA		NA	NA		NA	NA		NA	NA		NA
Race^d^														
Black	0.18 (0.08 to 0.28)	<.001	−0.08 (−0.15 to 0.00)		.05	−0.06 (−0.12 to 0.01)		.10	−0.02 (−0.09 to 0.04)		.53	−0.08 (−0.15 to −0.01)		.02
White	[Reference]	NA	[Reference]		NA	[Reference]		NA	[Reference]		NA	[Reference]		
Other^e^	0.22 (0.03 to 0.41)	.02	−0.15 (−0.30 to 0.00)		.04	−0.02 (−0.15 to 0.12)		.81	−0.06 (−0.18 to 0.06)		.31	−0.08 (−0.20 to 0.05)		.22
>2 Races	0.14 (−0.07 to 0.35)	.18	0.04 (−0.12 to 0.20)		.62	0.05 (−0.11 to 0.21)		.53	−0.08 (−0.23 to 0.06)		.27	0.00 (−0.14 to 0.14)		.97
Ethnicity^d^														
Hispanic	[Reference]	NA	[Reference]		NA	[Reference]		NA	[Reference]		NA	[Reference]		
Non-Hispanic	−0.23 (−0.32 to −0.13)	<.001	0.13 (0.06 to 0.20)		<.001	0.02 (−0.05 to 0.08)		.65	0.06 (0.00 to 0.12)		.07	0.01 (−0.05 to 0.07)		.77
Age														
18-29	[Reference]	NA	[Reference]		NA	[Reference]		NA	[Reference]		NA	[Reference]		
30-44	0.03 (−0.11 to 0.16)	.71	0.03 (−0.07 to 0.13)		.59	−0.08 (−0.17 to 0.01)		.09	−0.03 (−0.12 to 0.06)		.55	0.01 (−0.08 to 0.10)		.81
45-59	−0.09 (−0.21 to 0.04)	.20	−0.06 (−0.16 to 0.03)		.20	−0.03 (−0.12 to 0.06)		.53	−0.04 (−0.13 to 0.05)		.38	−0.01 (−0.10 to 0.08)		.77
>60	−0.09 (−0.22 to 0.03)	.14	−0.08 (−0.17 to 0.02)		.11	−0.08 (−0.17 to 0.01)		.08	−0.02 (−0.11 to 0.06)		.58	0.03 (−0.06 to 0.11)		.58
Political ideology														
Liberal	[Reference]	NA	[Reference]		NA	[Reference]		NA	[Reference]		NA	[Reference]		
Moderate	−0.11 (−0.20 to −0.02)	.02	−0.07 (−0.14 to 0.00)		.04	0.01 (−0.05 to 0.07)		.74	−0.02 (−0.08 to 0.04)		.52	−0.01 (−0.07 to 0.05)		.67
Conservative	−0.20 (−0.30 to −0.10)	<.001	−0.05 (−0.12 to 0.03)		.22	−0.08 (−0.15 to −0.02)		.01	0.05 (−0.02 to 0.11)		.17	−0.04 (−0.10 to 0.03)		.27
Household income, $														
<24 999	[Reference]	NA	[Reference]		NA	[Reference]		NA	[Reference]		NA	[Reference]		
25 000-49 999	0.11 (−0.02 to 0.25)	.10	0.02 (−0.09 to 0.12)		.77	−0.09 (−0.18 to 0.00)		.06	0.01 (−0.08 to 0.09)		.89	0.00 (−0.09 to 0.09)		.99
50 000-99 999	−0.19 (−0.31 to −0.06)	.004	0.11 (0.02 to .20)		.02	0.04 (−0.04 to 0.13)		.31	0.10 (0.02 to 0.18)		.01	0.05 (−0.03 to 0.13)		.24
>100 000	−0.12 (−0.25 to 0.01)	.07	0.18 (0.09 to 0.28)		<.001	−0.02 (−0.11 to 0.07)		.64	0.08 (0.00 to 0.17)		.05	0.06 (−0.02 to 0.15)		.12

Abbreviation: NA, not applicable.

^a^ This table reflects a single model with all interaction effect size estimates between each privacy protection and each demographic characteristic.

^b^ Differences are from a single full model with all interactions included.

^c^ Differences are from coefficients for interactions between each privacy protection and each demographic variable in the single full model.

^d^ Participants completed a general informed consent process followed by participant self-reporting race and ethnicity using the US Census Bureau categories.

^e^ Other includes Asian, American Indian, and Hawaiian and Pacific Islander.

## Discussion

This study has 3 main findings. First, consumers’ willingness to share personal health information varied considerably by contextual factors. The purpose of data use mattered most to consumers compared with any single privacy protection. Compared with research uses of their information, consumers were less willing to share data for clinical purposes and even less so for marketing purposes. Consumers seemed to be less sensitive to the particular entity using the data although less willing to share data with digital technology companies compared with university hospitals. These findings confirm prior literature on the importance of contextual factors and consumers’ preference for sharing health data for research purposes and clinicians.^32,33,34,35^

Second, consumers viewed consent as the most important privacy protection. The central role of consent may reflect the value placed by consumers on preserving autonomy and the ability to choose whether and how their personal data are used.^36^ These results affirm the importance of establishing consent as a baseline model of promoting digital data privacy. Moreover, each of the nonconsent protections, including ability to delete data, regulatory oversight, and transparency, was associated with an increased willingness to share data. Whereas none of the nonconsent protections were individually more important than consent, together the nonconsent protections were at least as important as consent alone, suggesting that a combination of consent with all the other nonconsent protections may maximize utility and consumer willingness to share their data. We did not find any evidence that combining protections was more than additive in their association with consumer utility. These results are consistent with a prior study^37^ that found fewer consumer privacy concerns when protections were viewed as being stronger. The present findings add to this literature by providing evidence on the relative importance of specific privacy protections beyond consent.

Third, willingness to share differed by sociodemographic characteristics. However, the importance of privacy protections varied little across subgroups. Overall, older and more conservative respondents were less willing to share health data. These findings are consistent with prior research^23^ demonstrating that older individuals are less likely to feel they have control over their digital information and less likely to believe they benefit from data governments collect from them. In contrast, political ideology has varying associations with digital privacy views and appears to be issue-dependent.^26^ For instance, people with conservative views expressed greater support relative to people with liberal views for domestic security purposes^38^ but were less supportive of using digital tools to reduce COVID-19 transmission.^21,39^ In addition, we found that although willingness to share was consistent across different racial groups, consent was more important to high-income White respondents. In addition, Hispanic compared with non-Hispanic respondents were more willing to share health data. In a prior study,^40^ minority groups, including Hispanic adults, have expressed greater concern about online privacy and security, but also have reported greater control over their health information from the internet relative to White respondents.^23^ Differences by ethnicity in the present findings may be due to the specific conjoint scenario, though further exploration is needed to better understand the reasons.

A key finding from this study, that many consumers would rather not share their digital health information when privacy protections are lacking but are more willing to share when more comprehensive privacy protections are established, points to the need to update and fill gaps in US privacy law. For example, the recent US Department of Health and Human Services guidance to protect patient privacy given the *Dobbs v Jackson Women’s Health* decision continues to rely on an outdated HIPAA framework without acknowledging the sensitive data generated from non–health related sources and without mentioning privacy protections.^41^ Ensuring comprehensive privacy protections may also benefit the users of the digital data by addressing consumers’ concerns and encouraging further data sharing. Although the European Union enacted digital privacy regulations in 2018 and in 2021 encompassing health and consumer digital information, the US has not. The California Consumer Privacy Act of 2020^42^ strengthened some aspects of consent, including allowing consumers the ability to opt out of data uses, but it continues to rely primarily on consent as a privacy protection.

Notably, consumers’ preferences in this study showing a low willingness to share digital health information for marketing purposes contrasts with their actual behavior as they frequently click through companies’ privacy agreements with limited privacy protections.^14^ Several potential reasons explain this contradiction, including that the agreements are often cumbersome to read and privacy protections difficult to understand; the desired product or service is more appealing than the potentially lost privacy; and the responses in our study based on hypothetical scenarios may not be trustworthy. There is also evidence that consumers care about their data privacy but simultaneously carry a sense of resignation about their control over use of their data.^43^ The reasons for these differences require more investigation, though the inconsistency also points to the need for rectifying the current model of obtaining consent and strengthening privacy protections.

Given the growing complexities of data sharing, unpredictable future uses of data, and the infeasibility of repeatedly acquiring consent for new uses, one approach to protecting consumer privacy is to implement a combination of individualized and early consent with collective and ongoing governance.^44,45^ Such a model would reduce individual burden while maintaining protections. Moreover, transparency and comprehensibility must apply both to the specific data being shared as well as how the data were collected and used.^16^ Ensuring a frictionless and efficient combination of privacy protections is vital to affirming cross-sectoral protection of consumer data, advancing regulation to meet twenty-first century needs, and leading to social progress by continually learning from data generated in a responsible manner.

### Limitations

This study has limitations. First, we included only 4 privacy protections, and others may be important to consumers. However, the inclusion of these specific protections was based on well-established privacy principles.^18^ Second, we did not ascertain whether respondents had a history of diabetes or provided care for someone with diabetes, which may affect their perceptions of data use for diabetes care. Third, our findings rely on ratings of hypothetical scenarios vs actual decisions, which may have resulted in different responses. However, conjoint analysis is a rigorous and well-established approach to measure preferences and individuals’ assessments of trade-offs and to estimate consumer decisions.^46,47^ Fourth, this study uses a cross-sectional design, and thus the findings reflect a particular moment in time when the survey was conducted in July 2020. The increase in use of digital platforms to reduce public harm from COVID-19, for example, may have affected findings. Fifth, similar to all survey findings, there may be important differences between responders and nonresponders. However, our survey had a relatively high response rate, and the conjoint experimental design allows for strong internal validity.

## Conclusions

In this national survey study using conjoint analysis, consumers’ willingness to share personal digital health information for health purposes was associated with the presence of specific privacy protections beyond consent alone. Additional protections, including data transparency, oversight, and data deletion may strengthen consumer trust and support socially beneficial uses of digital health data.

## Notes

Supplement 1. **eMethods.** Conjoint Survey Instrument **eTable.** Main Effects + Second Order Interactions

Supplement 2.
Data Sharing Statement
