# Supplementary material for: Consumer Views on Privacy Protections and Sharing of Personal Digital Health Information
Source: JAMA Netw Open. 2023 Mar 2;6(3):e231305. doi: 10.1001/jamanetworkopen.2023.1305 (PMC9982693; doi:10.1001/jamanetworkopen.2023.1305)
Supplement: Supplement 1. — eMethods. Conjoint Survey Instrument eTable. Main Effects + Second Order Interactions [file jamanetwopen-e231305-s001.pdf]

Supplemental Online Content

Gupta R, Iyengar R, Sharma M, et al. Consumer views on privacy protections and sharing of personal digital health information. *JAMA Netw Open.* 2023;6(3):e231305. doi:10.1001/jamanetworkopen.2023.1305

eMethods. Conjoint Survey Instrument

eTable. Main Effects + Second Order Interactions

This supplemental material has been provided by the authors to give readers additional information about their work.

© 2023 Gupta R et al. *JAMA Network Open*.

eMethods. Conjoint Survey Instrument

Base: All respondents

Intro [Display]

This survey is being conducted by the University of Pennsylvania. We want to learn your feelings about how digital information is collected and used. The questions should only take 25 minutes. All of your responses will remain completely confidential. Let's get started.

Scripting note: For each respondent, randomly select 15 rows from the design tab in "Conjoint1_Design.xlsx", insert text in conjoint based on values for each column within the each row. loop Conjoint1_x 15 times, each iteration with an insertion pattern based on lookup values in "Conjoint1_Design.xlsx". RANDOMIZE ORDER OF THE 15 QUESTIONS AND RECORD

Base: All respondents

Conjoint1_Intro [Display]

INTRODUCTION

Most Americans use digital technology in their daily lives. When you use digital technology, like your cell phone or computer, you leave information about yourself online. Experts believe that most of the information you leave behind can reveal something about your health. It is often possible to identify who you are from the information you leave behind.

This health information has a lot of possible uses. We are interested in what you think about these uses.

You will be shown **15 possible uses** of your digital information. In each case, you will be shown **who** will use the information, **what** information will be used, and **why** they are using it. Please indicate how willing you would be to share your health information for each situation.

Because each situation is different, please read each one carefully before providing your answer. We appreciate your honest opinions.

Base: All respondents

Conjoint1_x [Conjoint]

Please imagine you were asked to share your information in the following situation:

Who will use your information: *[INSERT USER VALUE BASED Lookup "Conjoint1_Design.xlsx" with insertion texts in "Insertion_Reference" tab]*
What information will be used: *[INSERT INFO VALUE BASED Lookup "Conjoint1_Design.xlsx" with insertion texts in "Insertion_Reference" tab]*
What they will use it for: *[INSERT USE VALUE BASED Lookup "Conjoint1_Design.xlsx" with insertion texts in "Insertion_Reference" tab] [INSERT DISEASE VALUE BASED Lookup "Conjoint1_Design.xlsx" with insertion texts in "Insertion_Reference" tab]*

How likely would you be to share your digital information in this situation?

© 2023 Gupta R et al. *JAMA Network Open*.

1. Definitely WOULD share
2. Probably WOULD share
3. Unsure
4. Probably WOULD NOT share
5. Definitely WOULD NOT share

Base: All respondents

Q1 [S]

In general, how would you rate your health?

1. Excellent
2. Very good
3. Good
4. Fair
5. Poor

Base: All respondents
RANDOMDIZE AND RECORD

Q3 [M]

Which type of apps do you currently have on your digital devices (computer, tablets, phones, etc.)?

1. News apps (local news, national headlines, technology announcements, etc.)
2. Navigation apps (maps, public transit)
3. Sports apps (sports schedules, scores, headlines, etc.)
4. Social networking apps (location check-ins, friend status updates, social media posts, etc.)
5. Entertainment apps (music, movies, television.)
6. Weather apps (local forecasts, natural disaster updates, etc.)
7. None of the above **[Exclusive, anchored]**

Base: All respondents

Q4 [S]

In general, digital technology like apps on your phone or computer can provide convenience but in exchange you sometimes give up privacy. Do you consider convenience or privacy more important?

1. Convenience is a lot more important than privacy
2. Convenience is a little more important than privacy
3. Neutral
4. Privacy is a little more important than convenience
5. Privacy is a lot more important than convenience

© 2023 Gupta R et al. *JAMA Network Open*.

Scripting note: For each respondent, randomly select 9 rows from the design tab in “Conjoint2_Design.xlsx”, insert text in conjoint based on values for each column within the each row. loop Conjoint2_x 9 times, each iteration with an insertion pattern based on lookup values in “Conjoint2_Design.xlsx”. RANDOMIZE ORDER OF THE 9 QUESTIONS AND RECORD.

Base: All respondents

Display2 [Display]
Next, we would like to ask you about some other uses of digital information. Please read the description of each situation and then tell us whether you approve or disapprove.

Base: All respondents

Conjoint2_x [Display]
[INSERT USER VALUE BASED Lookup “Conjoint2_Design.xlsx” with insertion texts in “Insertion_Reference” tab] used **[INSERT SOURCE VALUE BASED Lookup “Conjoint2_Design.xlsx” with insertion texts in “Insertion_Reference” tab]** to **[INSERT USE VALUE BASED Lookup “Conjoint2_Design.xlsx” with insertion texts in “Insertion_Reference” tab]**. They **[IF USE=1, INSERT “**published their results in a medical journal so that doctors could learn how to improve diabetes care**”][IF USE=2, INSERT, “**used this information to recommend changes to patients to improve their diabetes care
”][IF USE=3, INSERT “used this information to develop a marketing campaign to double the number of people taking a diabetes medication**”]**.

- ◊ **[IF CONSENT=1, INSERT “**People were asked permission for their information to be used.**”]**
- ◊ **[IF DATA=1, INSERT “**People were able to view the data that was collected from them.**]”**
- ◊ **[IF OVERSIGHT=1, INSERT “**A group of experts determined that personal privacy would be well-protected.**”]**
- ◊ **[IF DATADELETION=1, INSERT “**People could request that their data be erased at any time.**”]**

Do you approve or disapprove of how consumer digital information was used?

1. Strongly approve
2. Approve
3. Neutral
4. Disapprove
5. Strongly disapprove

Base: All respondents

Q2 [S]

© 2023 Gupta R et al. *JAMA Network Open*.

In the past 2 years, have you been the victim of identity theft, credit card theft or had other information stolen on the internet?

1. Yes
2. No
3. Not sure

Base: All respondents
RANDOMIZE AND RECORD

Q5 [Grid]
Next, we are going to name some institutions, companies and organizations that might collect and use digital health information from you. How confident are you that they will use your digital health information responsibly?

Statement in rows:

1. University hospitals
2. Doctors' offices
3. Genetic testing companies that you can pay to learn about your ancestry and genetic conditions
4. Pharmaceutical companies
5. Health insurance companies
6. Google
7. Apple
8. Facebook
9. FitBit
10. Your cell phone company
11. American Cancer Society
12. Local public health department run by your county or town
13. Your state health department
14. Federal government
15. Centers for Disease Control and Prevention
16. National Institutes of Health

Answer in columns:

1. Very confident
2. Moderately confident
3. Somewhat confident
4. Slightly confident
5. Not at all confident

© 2023 Gupta R et al. *JAMA Network Open*.

Scripting note: Split onto 2 screens with 8 items each.

CREATE DOV_Q6_AB:
Randomly assign 1 or 2 to each respondent

CREATE Q6_ITEM:

1	Vaccine use
2	Cancer (like breast or prostate cancer)
3	Problems with reproductive health (like infertility or erectile dysfunction)
4	Cough and cold symptoms
5	Lung diseases (like emphysema or asthma)
6	Joint problems or musculoskeletal pain
7	Fatigue
8	Stomach and gastrointestinal problems (like acid reflux or colitis)
9	Blood clots or bleeding disorders
10	Fungal infections of the skin
11	Physical disabilities
12	Developmental problems and disabilities
13	Heart problems (like heart attacks or high blood pressure)
14	Visual problems
15	Ear, nose and throat problems (like sinus or hearing issues)
16	Physical injuries (like falls or accidents)
17	Tobacco and alcohol use
18	Hormone and metabolism issues (like thyroid conditions, osteoporosis, or diabetes)
19	Kidney and urinary problems (like urinary tract infections or urinary incontinence)
20	Eating and nutrition habits
21	Mental health problems (like depression and anxiety)
22	Birth, pregnancy and breastfeeding issues
23	Neurologic problems (like seizures)
24	Sleep problems
25	Skin problems (like rashes, itching or excess sweating)
26	Obesity and weight issues
27	Genetic conditions that are transmitted from parent to child
28	Health insurance issues (like type of coverage or gaps in coverage)
29	COVID-19 or coronavirus infection
30	Allergies
31	Women's health issues (like menopause or menstrual symptoms)
32	Infectious illness or symptoms (like fever or meningitis)
33	Oral health problems (like dry mouth or cavities)
34	Conditions predominantly affecting children (like bedwetting)

© 2023 Gupta R et al. *JAMA Network Open*.

CREATE DOV_Q6_SELECT1-6:
Select 6 items from the 1-34 items in Q6_ITEM, each selected item gets recorded in DOV_Q6_SELECT1-6 respectively

Base: DOV_Q6_AB=1

Q6A [S]
How important is it to you to keep information about the following health issues private from companies or organizations when you use the internet or your smartphone?

Statement in rows:

1. **[INSERT DOV_Q6_SELECT1]**
2. **[INSERT DOV_Q6_SELECT2]**
3. **[INSERT DOV_Q6_SELECT3]**
4. **[INSERT DOV_Q6_SELECT4]**
5. **[INSERT DOV_Q6_SELECT5]**
6. **[INSERT DOV_Q6_SELECT6]**

Answers in columns:

1. Very Important
2. Moderately Important
3. Somewhat Important
4. Slightly Important
5. Not Important

Base: DOV_Q6_AB=2

Q6B [S]
How comfortable would you be with a marketing company using your online activity and interest in the following health topics to show you online advertisements?

Statement in rows:

1. **[INSERT DOV_Q6_SELECT1]**
2. **[INSERT DOV_Q6_SELECT2]**
3. **[INSERT DOV_Q6_SELECT3]**
4. **[INSERT DOV_Q6_SELECT4]**
5. **[INSERT DOV_Q6_SELECT5]**
6. **[INSERT DOV_Q6_SELECT6]**

Answers in columns:

1. Very comfortable
2. Moderately comfortable
3. Somewhat comfortable
4. Slightly comfortable
5. Not comfortable

CREATE DOV_RAND:
Assign 1 or 2 to each respondent

© 2023 Gupta R et al. *JAMA Network Open*.

Base: All respondents

COVIDDIS [Display]
The COVID-19 or coronavirus pandemic has caused a national public health emergency. Hospitals, doctors and public health officials are working to prevent the spread of disease. There are some ways that digital technology could be used to reduce the spread of disease. We are interested in your views about the use of digital technology to try to prevent the spread of COVID-19 infections. Please read about some possible uses and share your views about each.

Base: All respondents

QINDPOP1 [S]
When **[IF DOV_RAND=1, INSERT** "you"**][IF DOV_RAND=2, INSERT** "people"**]** use **[IF DOV_RAND=1, INSERT** "your phone"**][IF DOV_RAND=2, INSERT** "their phones"**]** or apps on **[IF DOV_RAND=1, INSERT** "your phone or computer"**][IF DOV_RAND=2, INSERT** "their phones or computers"**]** like social media, it is sometimes possible to detect early signs of COVID-19. This may be from changes in physical activity, social media postings, or other things like internet searches.

Do you agree or disagree with information from **[IF DOV_RAND=1, INSERT** "your phone or computer"**][IF DOV_RAND=2, INSERT** "people's phones or computers"**]** being used to monitor for COVID-19 symptoms so that public health officials can use that information to adjust public health policies like limits on large gatherings?

1. Strongly agree
2. Agree
3. Neither agree nor disagree
4. Disagree
5. Strongly disagree

Base: All respondents

QINDPOP2 [S]
Do you agree or disagree with this information from **[IF DOV_RAND=1, INSERT** "your phone or computer"**][IF DOV_RAND=2, INSERT** "people's phones or computers"**]** being shared with public health officials so they can contact **[IF DOV_RAND=1, INSERT** "you"**][IF DOV_RAND=2, INSERT** "people"**]** for testing if **[IF DOV_RAND=1, INSERT** "you"**][IF DOV_RAND=2, INSERT** "they"**]** have signs of COVID-19?

1. Strongly agree
2. Agree
3. Neither agree nor disagree
4. Disagree
5. Strongly disagree

© 2023 Gupta R et al. *JAMA Network Open*.

Scripting note: Display on the same page as QINDPOP1

Base: All respondents

QINDPOP3 [S]

"Smart" thermometers are thermometers connected to the internet. When **[IF DOV_RAND=1, INSERT** "you"**][IF DOV_RAND=2, INSERT** "people"**]** check **[IF DOV_RAND=1, INSERT** "your"**][IF DOV_RAND=2, INSERT** "their"**]** temperature with a smart thermometer to see if **[IF DOV_RAND=1, INSERT** "you"**][IF DOV_RAND=2, INSERT** "they"**]** have a fever, the thermometer sends that information to the company that makes them so they can track areas where people may be getting sick with COVID-19.

Do you agree or disagree that companies that make these thermometers should share **[IF DOV_RAND=1, INSERT** "your"**][IF DOV_RAND=2, INSERT** "people's"**]** fever information with public health officials so they can use that information to set public health policies like limits on large gatherings?

1. Strongly agree
2. Agree
3. Neither agree nor disagree
4. Disagree
5. Strongly disagree

Base: All respondents

QINDPOP4 [S]

Do you agree or disagree that companies that make these thermometers should share **[IF DOV_RAND=1, INSERT** "your"**][IF DOV_RAND=2, INSERT** "people's"**]** fever information with public health officials so **[IF DOV_RAND=1, INSERT** "you"**][IF DOV_RAND=2, INSERT** "they"**]** can be contacted for testing if **[IF DOV_RAND=1, INSERT** "you"**][IF DOV_RAND=2, INSERT** "they"**]** have a fever?

1. Strongly agree
2. Agree
3. Neither agree nor disagree
4. Disagree
5. Strongly disagree

Scripting note: Display on the same page as QINDPOP3

Base: All respondents

QINDPOP5 [S]

When a person has COVID-19, they can spread the disease even before they develop symptoms. One way to prevent spread of the disease is to contact all of the people the

© 2023 Gupta R et al. *JAMA Network Open*.

person was in contact with around the time they got sick and test and monitor those people. This is called contact tracing.

Apple and Google plan to launch a contact tracing program that would:

- Allow **[IF DOV_RAND=1, INSERT** "your cell phone"**][IF DOV_RAND=2, INSERT** "people's cell phones"**]** to keep track of who **[IF DOV_RAND=1, INSERT** "you"**][IF DOV_RAND=2, INSERT** "they"**]** have been around.
- Public health officials would send data to **[IF DOV_RAND=1, INSERT** "your phone"**][IF DOV_RAND=2, INSERT** "their phones"**]** each day so **[IF DOV_RAND=1, INSERT** "your phone"**][IF DOV_RAND=2, INSERT** "their phones"**]** could alert **[IF DOV_RAND=1, INSERT** "you"**][IF DOV_RAND=2, INSERT** "them"**]** if **[IF DOV_RAND=1, INSERT** "you"**][IF DOV_RAND=2, INSERT** "they"**]** had been in close contact with someone diagnosed with COVID-19.
- **[IF DOV_RAND=1, INSERT** "You"**][IF DOV_RAND=2, INSERT** "They"**]** could then get monitored and tested for COVID-19.

Do you agree or disagree with **[IF DOV_RAND=1, INSERT** "your cell phone"**][IF DOV_RAND=2, INSERT** "people's cell phones"**]** being used to determine if **[IF DOV_RAND=1, INSERT** "you"**][IF DOV_RAND=2, INSERT** "they"**]** come into close contact with someone diagnosed with COVID-19 so that **[IF DOV_RAND=1, INSERT** "you"**][IF DOV_RAND=2, INSERT** "they"**]** can be notified and get tested?

1. Strongly agree
2. Agree
3. Neither agree nor disagree
4. Disagree
5. Strongly disagree

Base: All respondents

QINDPOP6 [S]

Do you agree or disagree with **[IF DOV_RAND=1, INSERT** "your"**][IF DOV_RAND=2, INSERT** "people's"**]** information being provided to public health officials so they can contact **[IF DOV_RAND=1, INSERT** "you"**][IF DOV_RAND=2, INSERT** "them"**]** for testing and monitoring if **[IF DOV_RAND=1, INSERT** "you"**][IF DOV_RAND=2, INSERT** "they"**]** were exposed to COVID-19?

1. Strongly agree
2. Agree
3. Neither agree nor disagree
4. Disagree
5. Strongly disagree

Scripting note: Display on the same page as QINDPOP5

© 2023 Gupta R et al. *JAMA Network Open*.

Base: All respondents

QINDPOP7 [S]

For this program to work well and prevent COVID-19 spread, most people need to use it.

Do you agree or disagree that **[IF DOV_RAND=1, INSERT** "you"**][IF DOV_RAND=2, INSERT** "people"**]** should be required to participate in this program to protect **[IF DOV_RAND=1, INSERT** "yourself"**][IF DOV_RAND=2, INSERT** "themselves"**]** and other people?

1. Strongly agree
2. Agree
3. Neither agree nor disagree
4. Disagree
5. Strongly disagree

Base: All respondents

QINDPOP8 [S]

People with COVID-19 should be quarantined or kept home when they are sick to prevent the spread of disease. Some countries have used features on consumers' cell phones or other digital devices to enforce quarantines. This would mean tracking the location of people with COVID-19 to make sure they do not leave their home and expose others to COVID-19.

Do you agree or disagree with public health officials using features on **[IF DOV_RAND=1, INSERT** "your phone"**][IF DOV_RAND=2, INSERT** "people's phones"**]** to enforce a quarantine if **[IF DOV_RAND=1, INSERT** "you"**][IF DOV_RAND=2, INSERT** "they"**]** have COVID-19 to protect other people?

1. Strongly agree
2. Agree
3. Neither agree nor disagree
4. Disagree
5. Strongly disagree

Base: All respondents

QINDPOP9 [S]

Some countries have used information from people's phones or computers to determine if they are at high risk of having COVID-19 based on places and people they have been around. They have made phone apps that show if people are high risk. People who are high risk have been restricted from going public places, like using public transportation. This stops only the high risk people from going public places, instead of restricting everyone.

© 2023 Gupta R et al. *JAMA Network Open*.

Do you agree or disagree with public health officials using information from **[IF DOV_RAND=1, INSERT** "your phone or computer"**][IF DOV_RAND=2, INSERT** "people's phones or computers"**]** to determine if **[IF DOV_RAND=1, INSERT** "you"**][IF DOV_RAND=2, INSERT** "they"**]** are allowed to be in certain public places to protect other people?

1. Strongly agree
2. Agree
3. Neither agree nor disagree
4. Disagree
5. Strongly disagree

Base: All respondents
RANDOMIZE AND RECORD ITEMS

Q7 [Grid]
Cell phones could be used to keep track of whether you may have been around someone with COVID-19. Please tell us if you agree or disagree with the following statements:

Statement in rows:

1. People should be required to share information from their cell phone with public health officials if it means it can slow or stop the spread of COVID-19.
2. Cell phone companies should automatically put an app on people's phones that would allow people to know if they have been around someone with COVID-19 and notify them.
3. I want to share information from my cell phone with public health officials if it means it can slow or stop the spread of COVID-19.

Answer in columns:

1. Strongly agree
2. Agree
3. Neither agree nor disagree
4. Disagree
5. Strongly disagree

Base: All respondents
RANDOMIZE AND RECORD ITEMS

Q8 [Grid]
If digital technology like phones and computers are used to track COVID-19 exposures or infection, please rate your confidence in the following companies or organizations to protect this digital health information and make sure it is used responsibly:

Statement in rows:

1. Local public health department run by your county or town

© 2023 Gupta R et al. *JAMA Network Open*.

2. Your state health department
3. A health agency at the federal government
4. Apple
5. Google
6. Facebook
7. A company that makes digital thermometers
8. University researchers
9. Health insurance companies

<u>Answer in columns:</u>

1. Very confident
2. Moderately confident
3. Somewhat confident
4. Slightly confident
5. Not at all confident

Base: All respondents

Q9 [S]

Have you had COVID-19, or the illness caused by the novel coronavirus?

1. Yes – definitely
2. Yes – I think so
3. Maybe
4. No

Base: All respondents

Q10 [S]

Has a health care provider ever told you that you had COVID-19?

1. Yes – definitely
2. Yes – probably or suspected
3. No

Base: All respondents

Q11 [S]

Has an immediate family member been diagnosed with COVID-19?

1. Yes
2. No
3. Not sure

Base: All respondents
RANDOMIZE AND RECORD ITEMS

© 2023 Gupta R et al. *JAMA Network Open*.

Q12 [Grid]
How often are you taking the following actions to reduce your risk of exposure to COVID-19?

Statement in rows:

1. Washing hands frequently
2. Avoiding large gatherings
3. Avoiding contact with friends
4. Avoiding stores
5. Avoiding crowded public places
6. Wearing a face mask when around other people
7. Following government guidelines or rules to stay at home and limit contacts with other people

Answer in columns:

1. Almost Always
2. Very Often
3. Sometimes
4. Rarely
5. Never

Base: All respondents

Q13 [S]
In general, do you think of yourself as…

1. Extremely liberal
2. Liberal
3. Slightly liberal
4. Moderate, middle of the road
5. Slightly conservative
6. Conservative
7. Extremely conservative

Base: All respondents

PhoneConsent [S]
Researchers at the University of Pennsylvania would like to speak to you by telephone about your views on digital technology. The phone interview would take about 45 minutes, and you would be awarded **50,000 points ($50 equivalent)** for your time. If you agree to participate and consent to our providing your contact information to the researchers, the researchers might contact you in the next couple of weeks to schedule a telephone interview at a time convenient for you. Agreeing to be contacted does not guarantee you will be contacted, and if you are contacted, you will have the opportunity to decide whether or not you want to participate.

© 2023 Gupta R et al. *JAMA Network Open*.

If you're interested in participating in the phone interview for 50,000 points, please let us know what contact information we may give the researchers, so they can schedule the interview with you.

1. No, I don't want to participate in the phone interview; do NOT give my contact information to the researchers
2. Yes, I want to participate in the phone interview; you may give the following contact information to the researchers:
    a. First name (this must be provided if you want to participate in the phone interviews)
    b. Phone number
    c. Email address

Show KP closing question QF1

© 2023 Gupta R et al. *JAMA Network Open*.

eTable. Main effects + Second Order Interactions

**Data Attribute**	**Difference (95% CI)**	**P value**
Intercept	2.88 (2.8, 2.9)	<0.001
User		
Digital technology company	-0.17 (-0.20, -0.14)	<0.001
University hospital	[Reference]	
Source		
Health - EHR	0.08 (0.05, 0.10)	<0.001
Places you visit- Apps on your phone	[Reference]	
Use		
Clinical care	-0.09 (-0.12, - 0.06)	<0.001
Marketing	-0.40 (-0.43, -0.37)	<0.001
Research	[Reference]	
Privacy Protection		
Consent	0.41 (0.36, 0.46)	<0.001
Transparency	0.08 (0.03, 0.12)	0.001
Oversight	0.17 (0.12, 0.22)	<0.001
Data deletion	0.24 (0.19, 0.29)	<0.001
Interaction Terms between Attributes		
Consent x transparency	-0.02 (-0.07, 0.03)	0.44
Consent x oversight	-0.06 (-0.11, -0.01)	0.02
Consent x data deletion	-0.10 (-0.15, -0.05)	<0.001
Transparency x oversight	0.02 (-0.02, 0.07)	0.34
Transparency x data deletion	-0.01 (-0.05, 0.04)	0.83
Oversight x data deletion	-0.06 (-0.10, -0.01)	0.02

EHR, electronic health record

Source: Authors

© 2023 Gupta R et al. *JAMA Network Open*.
