# Supplementary material for: Consumer Views on Privacy Protections and Sharing of Personal Digital Health Information
Source: JAMA Netw Open. 2023 Mar 2;6(3):e231305. doi: 10.1001/jamanetworkopen.2023.1305 (PMC9982693; doi:10.1001/jamanetworkopen.2023.1305)
Supplement: Supplement 2. — Data Sharing Statement [file jamanetwopen-e231305-s002.pdf]

Data Sharing Statement

Gupta. Consumer Views on Privacy Protections and Sharing of Personal Digital Health Information. *JAMA Netw Open*. Published March 02, 2023. doi:10.1001/jamanetworkopen.2023.1305

Data
Data available: No

Additional Information
Explanation for why data not available: Data from this study can be shared upon request once ongoing analyses are complete.
